# Construction and Immunogenicity Evaluation of a Recombinant Fowlpox Virus Expressing VP2 Gene of African Horse Sickness Virus Serotype 1

**DOI:** 10.3390/microorganisms13122807

**Published:** 2025-12-09

**Authors:** Xiaohua Ma, Min Zhang, Xin Zhang, Ting Qi, Weiguo Zhang, Yang Zhao, Lei Na, Yingzhi Zhang, Xue-Feng Wang, Xiaojun Wang

**Affiliations:** 1 State Key Laboratory of Animal Disease Control and Prevention, Harbin Veterinary Research Institute, Chinese Academy of Agricultural Sciences, Harbin 150069, China; xiaohuama1299@163.com (X.M.); 13757169511@163.com (X.Z.); qiting2013@163.com (T.Q.); zweiguo00@163.com (W.Z.); zhaoyang0601@126.com (Y.Z.); nl2zy@163.com (L.N.); yingzhizhang1995@163.com (Y.Z.); 2 National Center for Veterinary Culture Collection, China Institute of Veterinary Drug Control, Beijing 102629, China; zmbooksea@sina.com; 3 Institute of Western Agriculture, Chinese Academy of Agricultural Sciences, Changji 831100, China

**Keywords:** African horse sickness virus, recombinant fowlpox virus, VP2, immunogenicity

## Abstract

African horse sickness (AHS) is a lethal vector-borne disease caused by African horse sickness virus (AHSV) and represents a major threat to equine health and the horse industry. In 2020, outbreaks of AHS caused by AHSV serotype 1 (AHSV-1) were reported in Thailand, increasing the risk of AHS introduction into China. Given the safety issues associated with currently available live attenuated AHS vaccines, the development of safer and more effective vaccination strategies is urgently needed. In this study, we constructed a recombinant fowlpox virus (rFPV) expressing the AHSV-1 VP2 protein as a candidate vaccine, designated rFPV-VP2. The recombinant virus was verified by PCR and Western blot analysis, which confirmed the successful expression of VP2. Preliminary immunization trials were conducted in both mice and horses, and immune responses were evaluated via an indirect enzyme-linked immunosorbent assay (iELISA) and immunofluorescence assay (IFA). The results revealed that VP2-specific antibodies were successfully induced in the serum of rFPV-VP2-immunized animals. Notably, serum from immunized horses showed specific reactivity with AHSV-1, confirming the induction of AHSV-1-specific immune responses. Therefore, these results demonstrate that rFPV-VP2 is a promising candidate vaccine for AHSV-1 and provide a scientific basis for the development of safer preventive strategies.

## 1. Introduction

African horse sickness (AHS) is a noncontagious, vector-borne infectious disease that affects equids and is characterized by alterations in respiratory and circulatory functions. All species in the Equidae family, including horses, mules, donkeys, and zebras, are susceptible to the disease. In susceptible populations of horses, mortality rates can exceed 90%. In contrast, mules and donkeys have been reported to demonstrate comparatively lower susceptibility [1,2,3,4]. Zebras are also markedly resistant with no clinical signs, except for fever [5]. The World Organization for Animal Health (WOAH) has listed AHS as a notifiable animal disease. In China, an AHS-free region, it has also been classified as a Class I infectious animal disease. The disease is prevalent mainly in sub-Saharan Africa, with sporadic outbreaks in North Africa, the Middle East, and Mediterranean countries [1,6]. Culicoides midges are the primary vector for AHS [7,8]. In 2020, Thailand reported its first AHS outbreak, followed by reports from Malaysia [9,10]. These events highlight the risk of AHS spreading to China and other neighboring countries [11].

The causative agent of the disease is the African horse sickness virus (AHSV), which belongs to Orbivirus in the subfamily Sedoreovirinae within the family Reoviridae. AHSV is a nonenveloped, segmented RNA virus. Its genome consists of 10 double-stranded RNA (dsRNA) segments encoding seven structural proteins (VP1–VP7) and four nonstructural proteins (NS1, NS2, NS3/3a, and NS4) [12,13]. Notably, VP2 is not only the most antigenically variable protein of AHSV but also the key determinant of its serotype. To date, nine distinct serotypes of AHSV (AHSV-1 to AHSV-9) have been identified on the basis of serum neutralization assays, and these serotypes show limited or no cross-neutralization [14,15].

Currently, there are no therapeutic treatments available for AHS [16,17]. A polyvalent live attenuated vaccine (LAV) against AHS, produced by Onderstepoort Biological Products (OBP, Pretoria), is licensed for use in South Africa and other African countries. However, LAV presents significant biosafety risks and is not approved in AHS-free countries [12]. LAVs have the potential to undergo genetic reassortment with field strains and revert to a virulent state [18]. These risks are particularly concerning in nonendemic regions, where the use of such vaccines remains controversial. Furthermore, another significant limitation of LAVs is their inability to differentiate infected animals from vaccinated animals [18]. An AHS inactivated vaccine was manufactured and used commercially in Spain, Portugal and Morocco between 1987 and 1991. Although it was proven to be effective at the time, this vaccine is no longer commercially available [12]. One of the key drawbacks of inactivated AHS vaccines is that they cannot differentiate between vaccinated and infected animals. Given the limitations of traditional vaccines in terms of safety, current efforts have increasingly focused on the development of recombinant vaccine platforms against AHS, including platforms such as subunit vaccines, DNA vaccines, virus-like particle (VLP) vaccines, and poxvirus-vectored vaccines [12].

Fowlpox virus (FPV) is a member of the genus Avipoxvirus (APV), which is in the subfamily Chordopoxvirinae of the Poxviridae family [19]. APV possesses a large DNA genome that can accommodate the insertion of foreign DNA with a relatively large genome size, and its replication is restricted to avian hosts, resulting in abortive infections only in mammalian hosts. Some APV-vectored veterinary vaccines have been developed for commercial use [20,21,22]. An attenuated FPV strain (S-FPV-017) was previously used as a vector to prepare avian and equine influenza virus vaccines [23,24]. VP2 is a major protective antigen of AHSV and is thus a key candidate for developing an AHS vaccine [25,26,27,28]. The present study aimed to develop an AHSV serotype 1 (AHSV-1) vaccine candidate by expressing the AHSV-1 VP2 protein using FPV as a viral vector. We successfully constructed, generated, and characterized the recombinant FPV expressing VP2 (rFPV-VP2). Subsequently, the immunogenicity of rFPV-VP2 was preliminary evaluated in both a model species (mice) and a target species (horses).

## 2. Materials and Methods

### 2.1. Cells, Viruses, Plasmids, and Animals

Primary chicken embryo fibroblast (CEF) cells were prepared following a previously described method [29]. These cells were cultured at 37 °C in a humidified incubator with 5% CO_2_ in Dulbecco’s modified Eagle’s medium (DMEM; Thermo Fisher Scientific, Waltham, MA, USA) supplemented with 10% heat-inactivated fetal bovine serum (FBS; Sigma–Aldrich, St. Louis, MO, USA) and antibiotics (0.1 mg/mL streptomycin and 100 IU/mL penicillin; Beyotime Biotechnology Co., Ltd., Nantong, China).

An attenuated FPV (S-FPV-017) was preserved in our laboratory [24]. The recombinant FPV expressing AHSV-1 VP2 (rFPV-VP2) was constructed on the basis of S-FPV-017 as a parental strain and propagated in CEFs via DMEM supplemented with 2% FBS.

With the laboratory-preserved plasmid pSY-HA-LacZ1 as the backbone [24], the equine influenza virus (EIV) hemagglutinin (HA) gene was replaced with the full-length VP2 gene of AHSV-1 (GenBank accession number: MT461278) to generate the FPV transfer vector pSY-VP2-LacZ1 via fusion PCR. The recombinant plasmid was verified by sequencing. The Cre recombinase expression plasmid pcDNA3-Cre was obtained from Addgene (Plasmid #198281).

Nine-day-old specific pathogen-free (SPF) fertilized eggs were provided by the State Resource Center of the Animal Laboratory for Poultry. Six-week-old specific pathogen-free (SPF)-grade BALB/c female mice (*n* = 15) were purchased from Liaoning Changsheng Biotechnology Co., Ltd. (Shenyang, China). At the end of the experiment, all mice were euthanized. Three healthy three-year-old Mongolian horses were raised on local farms in Harbin, China.

### 2.2. Generation of rFPV-VP2

rFPV-VP2 was generated as previously described [23,24,30]. Briefly, CEFs were infected with S-FPV-017, followed by transfection with the transfer vector pSY681-VP2-LacZ (2 μg) via Lipofectamine Reagent (Thermo Fisher Scientific). On the basis of the expression of the LacZ reporter gene, LacZ-positive (blue) plaques were selected via blue–white screening and subsequently purified through three rounds of continuous plaque cloning with X-gal staining. The purified recombinant virus rFPV-VP2-LacZ was identified via polymerase chain reaction (PCR) and Western blot (WB) analysis. To excise LacZ from rFPV-VP2-LacZ, CEF cells were transfected with 2 μg of pcDNA3-Cre, followed by infection with purified rFPV-VP2-LacZ at 24 h post-transfection (hpt). Three days later, LacZ-negative (white) plaques were picked and used to infect CEFs. Following five rounds of blue–white screening, rFPV-VP2 was further purified via a plaque assay with neutral red-supplemented agar in CEF cells, following a previously described method [31]. This was followed by identification through PCR and WB analysis, in which uninfected cells served as the blank control, while cells infected with S-FPV-017 were used as the negative control.

### 2.3. PCR

In this study, two pairs of PCR primers were designed to verify the successful construction of rFPV-VP2 and rFPV-VP2-LacZ (Table 1). Cellular DNA was extracted from CEF cells infected with rFPV-VP2, rFPV-VP2-LacZ or S-FPV-017 via the TIANamp Genomic DNA Kit (TIANGEN BIOTECH CO., LTD., Beijing, China) and then used as the PCR template. The PCR conditions were as follows: 95 °C for 5 min; then, 30 cycles of 95 °C (1 min), 52 °C (1 min), and 72 °C (3 min for VP2-F/VP2-R or 7 min for id F/id R); and a final extension at 72 °C for 10 min. The PCR products were analyzed via 0.8% agarose gel electrophoresis.

### 2.4. WB and Indirect Immunofluorescence (IFA)

WB and IFA were performed as previously described [31,32]. For WB, CEFs infected with rFPV-VP2 or S-FPV-017 were lysed in lysis buffer (50 mM Tris-HCl [pH 7.5], 50 mM NaCl, 5 mM EDTA and 1% Triton X-100) and subjected to electrophoresis on a 12% SDS–PAGE gel. An anti-VP2 monoclonal antibody (9E7) was used as the primary antibody [30], and anti-mouse IgG-DyLight 800 (Sigma–Aldrich, St. Louis, MO, USA; catalog number: SA5-35521), diluted 1:5000, was used as the secondary antibody.

In this study, IFA was employed to detect both the expression of VP2 in rFPV-VP2 and the serum antibody levels in horses immunized with rFPV-VP2:

For the detection of VP2 expression in rFPV-VP2, CEF cells were infected with either 0.1 MOI of rFPV-VP2 or 0.1 MOI of S-FPV-017 (as a specific control). At 96 h post-infection (hpi), the cells were processed as follows: washed three times with phosphate-buffered saline (PBS), fixed with 4% paraformaldehyde for 30 min, permeabilized with 0.1% Triton X-100 for 15 min, and finally blocked with 5% bovine serum albumin (BSA) in PBS for one hour. The cells were subsequently incubated with the 9E7 primary antibody for two hours, washed three times with PBS, and then incubated with FITC-conjugated goat anti-mouse IgG (whole molecule) (Sigma–Aldrich, USA; catalog number: 31547). The fluorescent signals were visualized via an EVOS M5000 inverted fluorescence microscope (Life Technologies, Bothell, WA, USA).

To detect the serum antibody levels in horses immunized with rFPV-VP2, Monolayer Vero cells cultivated in 96-well cell culture plates were infected with AHSV/C (an AHSV-1 strain) at a dose of 102.5 TCID_50_ per well in 0.1 mL [31]. Uninfected cells were used as a negative control. At 48 hpi, the cells were washed with PBS and fixed with cold methanol. Subsequently, 10-fold diluted serum from rFPV-VP2-immunized horses was added to the wells, and the plates were incubated at 37 °C for one hour. For the experimental controls, AHSV-1-positive horse serum (provided by the Pribright Institute, a WOAH-accredited AHS reference laboratory) was included as a positive control, whereas serum from horses before immunization with rFPV-VP2 was used as an additional negative control. After incubation, the serum was discarded, and the cells were washed three times with PBST (PBS containing 0.05% Tween-20), followed by two washes with PBS. After complete removal of the residual liquid, FITC-conjugated rabbit anti-horse IgG (H&L) (Sabbiotech; catalog number: L35083) was added, and the plates were incubated at 37 °C for one hour. Then, the cells were subjected to the same washing procedure as described above. Specific green fluorescent signals were observed via an OLYMPUS IX71 inverted fluorescence microscope.

### 2.5. Virus Titration

Monolayer CEFs cultured in 6-well plates were infected with serial dilutions of rFPV-VP2 or S-FPV-017 (1 mL per well), with five 10-fold serial dilutions ranging from 10^−1^ to 10^−5^. After adsorption at 37 °C for one hour, the inoculum was discarded, and the cells were washed three times with PBS. Each well was overlaid with a 1:1 mixture of 2% agarose (Sangon Biotech, China) and 2× virus growth medium. The plates were incubated at 37 °C with 5% CO_2_. Once cytopathic effects (CPEs) became visible via microscopy, a layer of neutral red agar was overlaid, followed by overnight incubation at 4 °C. On the following day, the number of plaques was visualized and counted at the appropriate dilutions. The plaque-forming units per milliliter (PFU/mL) were calculated via the following formula: PFU/mL = (number of plaques/inoculation volume) × dilution factor.

### 2.6. Animal Experiment

To evaluate the immunogenicity of rFPV-VP2, mice and horses were immunized with rFPV-VP2 in separate experiments.

For the mouse experiments, a total of 15 mice were randomly divided into three groups (*n* = 5 per group). Group 1 received an intramuscular inoculation of rFPV-VP2 at a dose of 10^6^ PFU per mouse. Group 2 served as the negative control and received an intramuscular inoculation of S-FPV-017 at the same dose (10^6^ PFU per mouse). Group 3 served as the blank control and received an intramuscular injection of DMEM (200 μL per mouse). Each group received two immunizations at three-week intervals. The mice were monitored daily for clinical signs over a 12-week period. Serum samples were collected from all mice at two-week intervals and subsequently subjected to ELISA to evaluate their reactivity to AHSV-1 VP2.

For the horse experiment, three clinically healthy adult horses were intramuscularly inoculated in the neck with 1 × 10^8^ PFU of rFPV-VP2 per horse. A booster immunization with the same dose and route was administered three weeks after the primary inoculation. The clinical signs of the horses were monitored daily for 12 weeks. Serum samples were collected from all horses and subjected to ELISA to evaluate their reactivity with AHSV-1 VP2. Additionally, the serum collected on day 14 after the second vaccination was subjected to IFA to assess its reactivity to AHSV-1.

### 2.7. Preparation of Recombinant AHSV-1 VP2 Protein

The C-terminal His-tagged AHSV-1 VP2 gene (GenBank accession number: MT461278) was cloned and inserted into the pFastBac vector to generate the pFastBac-VP2 recombinant plasmid. The plasmid was subsequently transformed into E. coli DH10Bac cells. Positive clones were identified through blue–white colony screening and confirmed by sequencing to verify the correct insertion of the target gene. Bacmid plasmids were then extracted from the positive E. coli colonies. A total of 2 μg of the recombinant Bacmid plasmid was transfected into Sf9 cells via the Lipofectamine™ 2000 reagent (Thermo Fisher Scientific). Sf9 cells were seeded at a density of 5 × 10^5^ cells per well and cultured in SF-900 II SFM (Gibco, Thermo Fisher Scientific), which is serum free and specifically formulated to meet the nutritional requirements of insect cells. The cultures were maintained at 27 °C to prevent overgrowth and minimize the accumulation of reactive oxygen species. Sterile conditions and proper aeration were ensured throughout the process. At 96 h post-transfection (hpt), the supernatant and cell lysates were harvested as the P1 generation recombinant virus. This P1 supernatant was then used to infect a fresh batch of Sf9 cells at a 2% inoculation ratio, with a cell density of approximately 2 × 10^6^ cells/mL. After 72 h of incubation, the supernatant (designated P2 generation) was collected and used to infect 100 mL of Sf9 cells at a 5% inoculation ratio. The recombinant AHSV-1 VP2 protein was purified as described previously [33,34], and its concentration was determined via a BCA protein assay kit (Beyotime).

### 2.8. Development of an Indirect ELISA (iELISA) Based on Recombinant AHSV-1 VP2 for Antibody Detection

The purified recombinant VP2 protein was diluted to a concentration of 300 ng/well with PBST and coated onto a 96-well microplate, followed by incubation overnight at 4 °C. After incubation, the plate was washed three times with PBST. Next, 200 μL of 5% skim milk in PBST was added to each well for blocking, and the plate was incubated at room temperature for 2 h. Following blocking, the plate was washed three times with PBST as described above. Serum samples were diluted 1:200 with PBST, and 100 μL of the diluted serum was added to each well. The plate was incubated at 37 °C for 1.5 h and then washed three times with PBST. Subsequently, 100 μL of HRP-conjugated goat anti-mouse IgG (H+L) (1:5000 dilution) or HRP-conjugated rabbit anti-horse IgG (H+L) (1:10,000 dilution) was added. The plate was incubated at 37 °C for 1 h, followed by three washes with PBST. Color development was initiated by adding 100 μL of TMB substrate solution to each well, and the mixture was incubated at room temperature for approximately 10 min in the dark. The reaction was terminated by adding 50 μL of 2 M H_2_SO_4_ to each well, and the absorbance was measured at 450 nm via a microplate reader. Sera from animals prior to rFPV-VP2 inoculation were used as negative controls. All samples were tested in triplicate.

### 2.9. Virus Neutralization (VN) Test

To assess the serum neutralizing antibody titers against AHSV-1 in horses inoculated with rFPV-VP2, VN tests were performed as previously described [31]. Briefly, 0.5 mL of AHSV/C (an AHSV-1 strain), diluted to 100 TCID50/0.1 mL, was mixed with an equal volume of serially diluted (twofold, fourfold and eightfold) horse serum from rFPV-VP2-inoculated animals. The mixtures were then incubated at 37 °C for 1 h before being inoculated into four replicate wells of a 96-well plate containing Vero cell monolayers, after which 0.1 mL of the mixture was added to each well. The plates were incubated at 37 °C and 5% CO_2_ for 120 h, and the number of wells with CPEs in each group was determined via an inverted microscope. AHSV-1-positive horse serum provided by the Pribright Institute was included as a positive control, and serum from a horse prior to inoculation with rFPV-VP2 was used as a negative control, with both subjected to the same dilution gradients.

## 3. Results

### 3.1. Construction of rFPV-VP2

**Figure 1:**
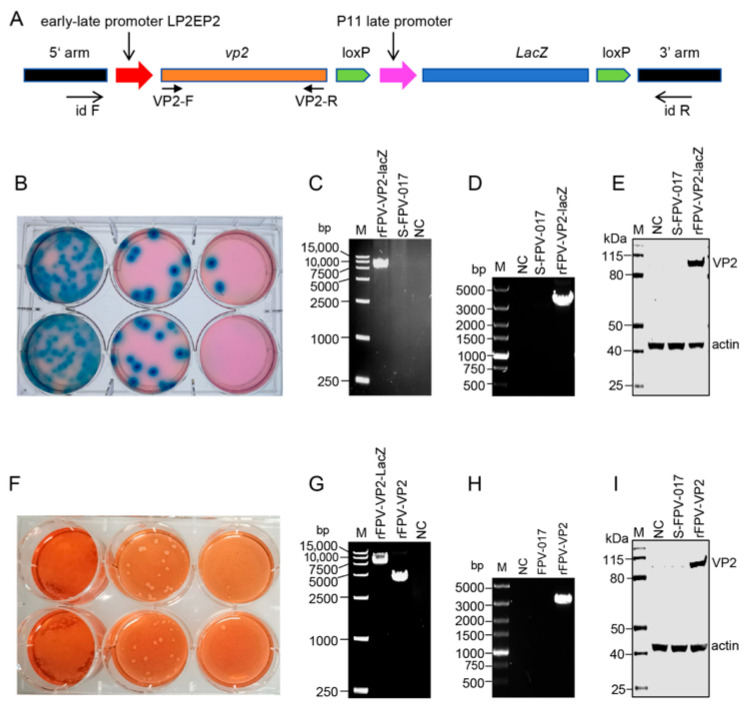
Construction and detection of the recombinant fowlpox virus containing AHSV-1 VP2. (**A**) Schematic representation of the recombinant plasmid pSY-VP2-LacZ1. The recombinant plasmid contains the VP2 gene with the LP2EP2 promoter and the LacZ reporter gene with the p11 promoter, and LacZ is flanked by loxP sites. (**B**) Plaques formed during the screening of the recombinant virus rFPV-VP2-LacZ; blue plaques indicate positive viral plaques following X-gal staining. (**C**,**D**) PCR identification of the recombinant rFPV-VP2-LacZ virus. rFPV-VP2-LacZ, CEFs infected with recombinant virus; S-FPV-017, parental virus control; NC (negative control), uninfected CEF cells; M, DNA marker. PCR amplification was performed via id-F/id-R primers located within the fowlpox virus homologous arms, revealing the insertion of both the VP2 gene and the LacZ reporter gene (>7 kb) (**C**). Specific amplification of the VP2 gene via the VP2-F/VP2-R primer produced a > 3 kb product (**D**). (**E**) WB analysis of lysates from rFPV-VP2-LacZ-infected CEFs via a VP2-specific antibody. rFPV-VP2-LacZ, CEF cells infected with recombinant virus; S-FPV-017, parental virus control; NC, uninfected CEFs. (**F**) Plaque purification of rFPV-VP2. White plaques were purified via low-melting-point agarose containing neutral red, and the white clear plaques confirmed the successful isolation of the recombinant virus. (**G**) PCR analysis confirmed the deletion of the LacZ gene from rFPV-VP2-LacZ. When id-F/id-R primers were used for PCR amplification, rFPV-VP2-LacZ generated a PCR product of approximately 7 kbp, whereas rFPV-VP2 produced a band of approximately 3 kb, indicating successful deletion of the LacZ gene. NC, negative control. (**H**) Using the VP2-F/VP2-R primers, the VP2 gene of rFPV-VP2 was specifically amplified, generating an amplicon of approximately 3000 bp. (**I**) WB analysis of lysates from rFPV-VP2-infected CEFs via a VP2-specific antibody. This experiment was performed three times, and a representative result is shown.

FPV has been used extensively as a delivery vector for various infectious diseases in humans and animals [35,36,37,38]. Previously, an equine influenza virus vaccine based on rFPV expressing HA was prepared in our laboratory, which could protect animals from challenge with the virus [24]. In this study, to develop an AHSV-1 candidate vaccine using FPV as a vector, we constructed an FPV transfer vector containing the full-length AHSV-1 VP2 gene, which was designated pSY-VP2-LacZ. Notably, the LacZ reporter gene in this vector is flanked by loxP sites, enabling subsequent excision of the reporter gene as needed (Figure 1A). Using this transfer vector, we rescued a recombinant virus carrying both VP2 and the LacZ reporter gene (rFPV-VP2-LacZ) via blue–white screening as previously described (Figure 1B) [23,24], with blue plaques selected to confirm successful isolation of rFPV-VP2-LacZ. The expression of VP2 in rFPV-VP2-LacZ-infected CEF cells was verified by PCR (Figure 1C,D) and WB (Figure 1E).

To avoid potential interference of LacZ with VP2 activity in subsequent experiments, the LacZ gene was excised from rFPV-VP2-LacZ via Cre recombinase, successfully yielding rFPV-VP2. The resulting rFPV-VP2 was subjected to five rounds of blue–white screening, and white plaques were selected for subsequent plaque purification to confirm successful isolation of rFPV-VP2 (Figure 1F). We validated the complete deletion of the LacZ gene via PCR (Figure 1G,H) and confirmed the expression of VP2 in rFPV-VP2-infected CEFs via WB (Figure 1I).

### 3.2. Replication Characteristics of rFPV-VP2

**Figure 2:**
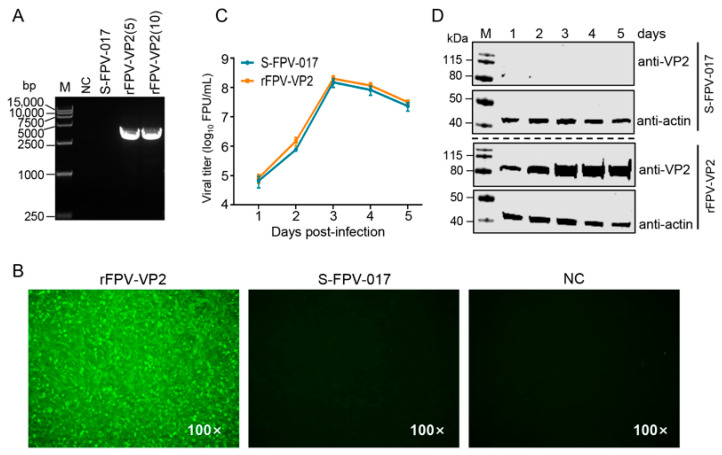
Replication kinetics and genetic stability of recombinant rFPV-VP2. (**A**) Following ten serial passages in CEF cells, PCR analysis confirmed that the VP2 gene was stably retained in rFPV-VP2. rFPV-VP2 (5) and rFPV-VP2 (10) represent the 5th and 10th generations of the virus, respectively; parental virus S-FPV-017 served as a specific control; NC (negative control), which represents uninfected CEFs, served as a negative control. (**B**) IFA confirmed stable expression of the VP2 protein in CEFs infected with rFPV-VP2. (**C**) Comparative growth curves of recombinant rFPV-VP2 and the parental virus (S-FPV-017) in CEFs. (**D**) Western blot analysis was performed to detect VP2 protein expression in CEFs infected with rFPV-VP2 and S-FPV-017 at the indicated time points. These experiments were performed three times, and a representative result is shown.

rFPV-VP2 was passaged 10 times in CEFs. PCR analysis demonstrated that the VP2 gene in rFPV-VP2 remained genetically stable during passage (Figure 2A). The expression of the VP2 protein was subsequently verified by IFA. Specific fluorescence was detected in CEFs infected with rFPV-VP2 via a VP2-specific antibody, whereas no fluorescence signal was detected in cells infected with parental S-FPV-017 (Figure 2B). These results confirm that rFPV-VP2 stably expresses VP2 in CEFs.

To evaluate whether VP2 expression influences viral replication, CEFs were infected with either rFPV-VP2 or the parental virus S-FPV-017 at an MOI of 0.1. Viral cultures were harvested every 24 h, and titers were determined at each time point. The viral growth curve revealed no significant differences in replication kinetics between the two viruses, and both reached a maximum titer of 10^8^ PFU/mL at 3 d post-infection (Figure 2C and Supplementary Table S1). This result also demonstrated that the insertion of the VP2 gene did not alter the growth properties of the parental S-FPV-017. Furthermore, WB analysis revealed that the expression of VP2 in rFPV-VP2-infected cells exhibited parallel fluctuations with respect to viral replication levels, indicating a consistent association between VP2 expression and viral propagation (Figure 2D and Figure S1).

### 3.3. Immunogenicity of rFPV-VP2 in Mice and Horses

**Figure 3:**
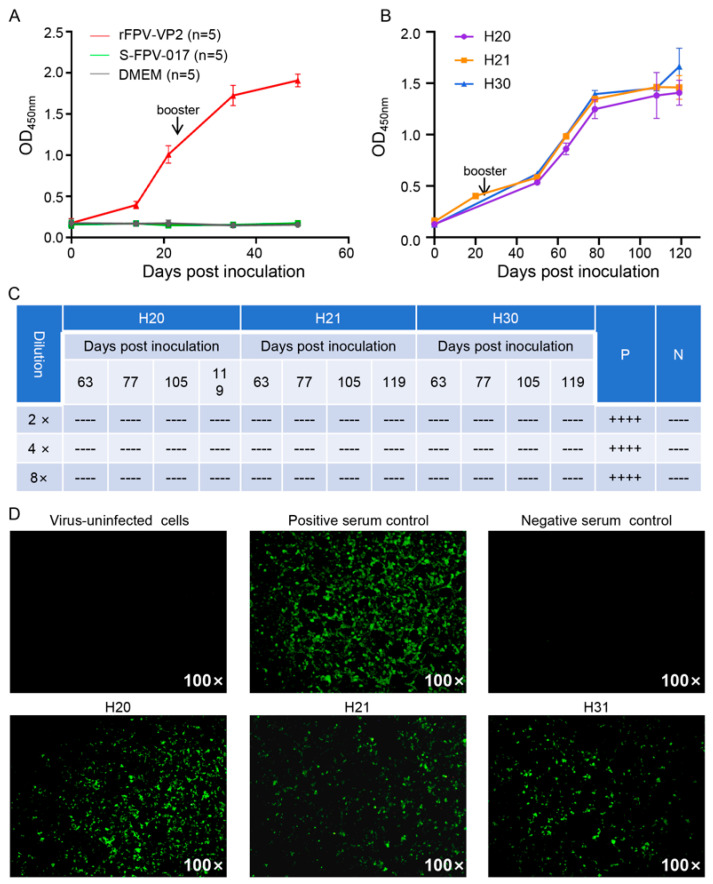
Antibody response induced by rFPV-VP2 in mice and horses. (**A**) Serum samples were collected from three groups of mice inoculated with rFPV-VP2, S-FPV-017, or DMEM at the indicated time points. Mean IgG antibody levels (five mice per group) were measured via iELISA, which was established on the basis of the baculovirus-expressed VP2 protein of AHSV-1. The 0 day represents the time point before inoculation. Booster refers to the time point of the second vaccination. (**B**) Serum samples were collected from three horses (designated H20, H21, and H31) inoculated with rFPV-VP2 at the indicated time points. IgG antibody levels in these serum samples were measured via the same iELISA as described in (**A**). The serum samples from H20 and H31, collected at 21 days post-primary vaccination, were missing and thus could not be analyzed. The 0 day represents the time point before inoculation. Booster refers to the time point of the second vaccination. (**C**) Serum samples collected from all three horses inoculated with rFPV-VP2 at the indicated time points were subjected to the VN test, with serial twofold, fourfold, and eightfold dilutions. Each sample was tested in four replicate wells, and the presence of a CPE in each well was recorded. A “-” was assigned to wells showing CPEs, and a “+” was assigned to those without CPEs. P, positive control; N, negative control. (**D**) Serum samples were collected 21 days post-booster vaccination, and antibodies against AHSV/C were detected via IFA. Sera from immunized horses (H20, H21, H31) were tested on AHSV/C-infected Vero cells. Positive control serum, negative control serum, and uninfected cells served as controls. The experiment was repeated three times, and representative images are shown.

To evaluate the immunogenicity of rFPV-VP2, five mice were immunized with rFPV-VP2 (immunization group), five with S-FPV-017 as a specific control (specific control group), and five with DMEM as a blank control (blank control group). Throughout the entire observation period (7 weeks), all mice showed no obvious clinical symptoms. Serum antibody detection via the VP2-based iELISA established in this study revealed that the IgG antibody titer in the immunization group gradually increased over time. Specifically, a moderate increase in the IgG titer was observed after the primary immunization, whereas a rapid and significant increase occurred following the booster immunization. In contrast, the serum IgG levels in both the specific control group and the blank control group remained low throughout the experiment, with no significant increase (Figure 3A and Supplementary Table S2). These results indicate that immunizing mice with rFPV-VP2 can induce the body to produce specific IgG antibodies against the VP2 protein.

To evaluate the immunogenicity of rFPV-VP2 in AHSV-targeted animals, three clinically healthy adult horses were inoculated intramuscularly with rFPV-VP2, followed by a booster dose 3 weeks later. Throughout the entire observation period (11 weeks), all horses showed no obvious clinical symptoms. Serum antibody detection via ELISA revealed that the IgG titers in all three horses gradually increased over time after immunization (Figure 3B and Supplementary Table S3), which was consistent with the antibody response trend observed in the rFPV-VP2-immunized mouse group. These results suggest that immunizing horses with rFPV-VP2 stimulates the production of specific IgG antibodies against VP2. To subsequently examine whether rFPV-VP2 can induce the production of serum neutralizing antibodies (nAbs), serum samples collected from all three horses at 28 days after the second immunization were tested via the VN test. Unfortunately, none of the samples were able to inhibit the development of CPE at any dilution (Figure 3C), indicating the absence of AHSV-1-neutralizing antibodies in these serum samples. In addition, IFA was performed to detect AHSV-1-infected Vero cells via serum samples collected from all three horses at 14 days post-booster immunization. Notably, serum from rFPV-VP2-immunized horses produced strong and specific fluorescence signals, indicating the presence of VP2-specific antibodies capable of recognizing AHSV antigens. No detectable fluorescence was detected in sera from preimmunized horses (Figure 3D). These results further demonstrate that rFPV-VP2 successfully induced specific antibody responses against AHSV-1 in horses, albeit without eliciting neutralizing antibodies.

## 4. Discussion

In recent years, rising global temperatures have created a warmer and more humid environment, providing vectors, such as mosquitoes and Culicoides midges (the vectors of AHS), with broader living spaces and longer active periods. There has been a sharp increase in cases of vector-borne diseases, such as dengue fever and chikungunya, worldwide [39,40]. Similarly, AHS, transmitted by insect vectors, such as *Culicoides* spp., results in high mortality in infected equids. Notably, an AHS outbreak caused by AHSV-1 in Thailand in 2020 posed a substantial threat to neighboring nations [11,41]. In light of the potential risk of introducing and spreading AHS in China, multiple research institutions in China have proactively initiated research projects, including the development of AHS detection methods and candidate vaccines, as well as the investigation of the biological characteristics and distribution of its vector (*Culicoides* spp.) [31,42,43,44].

AHSV VP2 is the main target for inducing neutralizing antibodies in host animals and is therefore a key focus in AHS vaccine research. Previous studies have shown that expressing AHSV-VP2 via baculovirus or vaccinia virus systems can effectively protect horses against lethal challenges, and most of these studies focused on AHSV-4 [45,46,47,48]. The general consensus is that attenuated vaccines for AHS are not suitable for use in nonendemic countries or zones because of biosafety concerns. In addition, neither attenuated nor inactivated vaccines can be used in strategies that differentiate between infected and vaccinated animals, a critical limitation for maintaining AHS-free status and accurate epidemiological monitoring. However, the VP2-based vaccine strategy can effectively overcome these limitations. The 2020 AHS outbreak in Thailand was the first AHS outbreak in Southeast Asia, as well as the first outbreak of AHSV-1 outside Africa. To address the potential threat posed by this emerging epidemic, the present study aimed to develop an AHSV-1 vaccine candidate, and we successfully constructed a recombinant FPV expressing AHSV-1 VP2, designated rFPV-VP2.

Poxviruses are widely used as key platforms in the development of equine vaccines, including those against equine influenza virus, West Nile virus, and equine herpesvirus infections [49,50,51]. This established utility, alongside emerging platforms such as precision-engineered mRNA vaccines and multi-epitope subunit vaccines [52,53], highlights the diverse technological approaches available for modern vaccine development. Specifically, FPV has been exploited as a safe, non-replicating vector for mammalian vaccination and is employed to prevent various diseases [19,37,38]. Previously, our laboratory constructed a recombinant FPV expressing the EIV HA protein. This recombinant FPV was shown to be not only safe for horses but also effective in protecting them against experimental EIV challenge [24]. In the present study, we first utilized this FPV vector to generate a recombinant FPV expressing AHSV-1 VP2, which contained the screening gene LacZ (designated rFPV-VP2-LacZ). The reporter gene LacZ is routinely introduced alongside exogenous genes to facilitate the screening of recombinant FPV during preparation. Additionally, we removed the LacZ gene from rFPV-VP2-LacZ via Cre recombinase, generating rFPV-VP2. This modification eliminates the potential risk of LacZ interfering with VP2 activity. After ten passages in CEFs, the replication levels of rFPV-VP2 were similar to those of its parental strain S-FPV-017, and stable expression of the VP2 protein was achieved. These results demonstrate the stability of rFPV-VP2. Furthermore, IFA confirmed that a VP2-specific monoclonal antibody could specifically recognize CEF cells infected with rFPV-VP2, indicating that rFPV-VP2 possesses favorable antigenicity.

To detect the antibody levels induced by rFPV-VP2 immunization in animals, we first prepared AHSV-1 VP2 protein expressed via insect baculovirus and established an iELISA using this recombinant protein. Detection via this method revealed that the immunization of mice and horses with rFPV-VP2 significantly increased the VP2-specific antibody levels. In the horse experiment, serum samples collected from all three immunized horses on day 21 postbooster immunization tested positive for AHSV-1 by IFA. However, due to experimental constraints, only samples from this time point were analyzed via IFA, with those from other time points remaining untested. Due to constraints related to experimental conditions and funding, only 3 horses were immunized with rFPV-VP2, and no control group immunized with S-FPV-017 was included, this represents a significant limitation when evaluating the vaccine’s immunogenicity. Nevertheless, we demonstrated that rFPV-VP2 can induce specific antibody production in horses by comparing pre- and post-immunization AHSV-1-specific serum antibody responses in these three horses. Thus, the data provide preliminary yet meaningful evidence of the immunogenicity of rFPV-VP2, supporting its use in initial exploratory studies. Numerous studies have identified serum neutralizing antibodies (nAbs) that target the VP2 protein as key protective factors against AHSV infection. Nevertheless, in the present study, VN tests revealed that no AHSV-1-neutralizing antibodies were detected in any of the rFPV-VP2-inoculated horses at the time point after the second immunization. This observation may be attributed to several factors. First, the expression level or conformational folding of the recombinant VP2 antigen in the rFPV vector may have been insufficient to expose key AHSV-1 neutralizing epitopes, thereby impairing the induction of functional neutralizing antibody responses. Second, the immunization protocol (e.g., inoculation dose or interval between immunizations) may have been suboptimal, failing to stimulate the humoral immune system adequately to generate detectable neutralizing antibodies. Third, individual immune heterogeneity among horses may have contributed to poor responsiveness to the rFPV-VP2 vaccine, as variations in MHC haplotypes or innate immune status can influence antibody production against viral antigens. Additionally, the small sample size of three horses in this study should not be overlooked. This limited number of animals may lack statistical representativeness, potentially masking variations in antibody responses among individuals or leading to biased results that do not reflect the broader horse population. A previous study reported that horses vaccinated with a recombinant vaccinia virus expressing AHSV-4 VP2 were protected against challenge with a virulent AHSV-4 strain, although this protection required three vaccine doses. Notably, low titers of nAbs were only detectable after the third inoculation, and these titers increased significantly after subsequent challenge with the virulent strain [48]. Another study reported that an NS3/NS3a-deficient attenuated AHSV-5 vaccine can confer protection despite inducing only extremely low nAb titers (<4) during the immunization phase; notably, following challenge with a virulent AHSV-5 strain, nAb production was still elicited in the vaccinated ponies [54]. Similar findings have also been reported in other studies focusing on AHS vaccines and this phenomenon of achieving protective efficacy without detectable high-titer nAbs is hypothesized to be closely associated with robust cellular immune responses, particularly those elicited by poxvirus vectors [55,56].

As China is an AHS-free country and our laboratory does not possess virulent AHSV strains, this study was unable to assess whether rFPV-VP2 immunization can induce protective immunity against AHSV challenge. Additionally, the immune characteristics induced by rFPV-VP2 immunization, including the dynamic changes in neutralizing antibody titers over time, T/B-cells response, and cytokine profile, remain to be further investigated. Further studies are needed to optimize the immunization strategy. For example, by adjusting the vaccine dosage, modifying the interval between inoculations, increasing the number of inoculations, assessing the need for adjuvants, and exploring and testing more efficient promoters. These studies would support a more detailed evaluation of the immunological characteristics of the rFPV-VP2 vaccine. Perhaps most importantly, we should pursue collaboration with international research institutions, particularly those in South Africa, a key endemic region for AHS, to jointly explore the protective efficacy of this candidate vaccine.

In summary, this study constructed an rFPV-VP2, which was shown to effectively induce AHSV-1 VP2-specific antibodies in horses. Although a detailed characterization of postvaccination immune responses in horses is lacking, rFPV-VP2 still serves as a promising candidate for subsequent research on AHSV-1 vaccines.

## Figures and Tables

**Table 1:** Primers used for the identification of rFPV-VP2 and rFPV-VP2-LacZ by PCR.

Primers	Sequence (5′–3′)	Description
VP2-F	GCGTCTGAATTTGGAATTCTATTGACC	Specific amplification of the full-length AHSV-1 VP2 gene (3171 bp).
VP2-R	CTCTATCTTCGACAATAACTTTGAGAAG	
id F	ATGAAATATTAGATTCTAGCGGTTGGTC	Targets the 5′ and 3′ homologous recombination arms of FPV; differentiates rFPV-VP2-LacZ (>7000 bp) from rFPV-VP2 (>4000 bp) based on PCR product size.
id R	ATCTCGTAAGATGCCTATATGAATATGG	

## Data Availability

The data presented in this study are openly available in GenBank at [MT461278], reference number [41].

## Supplementary Materials

The following supporting information can be downloaded at: https://www.mdpi.com/article/10.3390/microorganisms13122807/s1, Figure S1: Western blot analysis was performed to detect VP2 protein expression in CEFs infected with S-FPV-017 and rFPV-VP2 at the indicated time points. The entire western blot images corresponding to Figure 2D are provided; Table S1: Viral replication dynamics of S-FPV-017 and rFPV-VP2 in CEF cells; Table S2: Indirect ELISA detection of antibody responses to rFPV-VP2 in mouse serum; Table S3: Indirect ELISA detection of antibody responses to rFPV-VP2 in horse serum.

## Abbreviations

The following abbreviations are used in this manuscript:

AHSV	African horse sickness virus
AHS	African horse sickness
iELISA	Indirect enzyme-linked immunosorbent assay
CEF	chicken embryo fibroblast
IFA	Immunofluorescence assays
VN	Virus neutralization
rFPV	Recombinant fowlpox virus
