# Supplementary material for: Construction and Immunogenicity Evaluation of a Recombinant Fowlpox Virus Expressing VP2 Gene of African Horse Sickness Virus Serotype 1
Source: Microorganisms. 2025 Dec 9;13(12):2807. doi: 10.3390/microorganisms13122807 (PMC12735409; doi:10.3390/microorganisms13122807)
Supplement: Supplementary file 1 [file microorganisms-13-02807-s001.zip › Supplementary Table S1.pdf]

Supplementary Table S1. Viral replication dynamics of S-FPV-017 and rFPV-VP2 in CEF cells.

Group	Day post infection (virus titer, PFU/mL)				
	1	2	3	4	5
S-FPV-017	5×10^4	7.5×10^5	1.5×10^8	1×10^8	2.5×10^7
	5×10^4	8×10^5	2×10^8	1×10^8	1.5×10^7
	1×10^5	7.5×10^5	1×10^8	5×10^7	3×10^7
rFPV-VP2	5×10^4	1×10^6	2×10^8	1×10^8	3.5×10^7
	5×10^4	2×10^6	2.5×10^8	1×10^8	3.5×10^7
	5×10^4	1.5×10^6	1.5×10^8	1.5×10^8	2.5×10^7

Note: Three replicates were performed for each sample.
