# Supplementary material for: Construction and Immunogenicity Evaluation of a Recombinant Fowlpox Virus Expressing VP2 Gene of African Horse Sickness Virus Serotype 1
Source: Microorganisms. 2025 Dec 9;13(12):2807. doi: 10.3390/microorganisms13122807 (PMC12735409; doi:10.3390/microorganisms13122807)
Supplement: Supplementary file 1 [file microorganisms-13-02807-s001.zip › Supplementary Table S2.pdf]

Supplementary Table S2 Indirect ELISA detection of antibody responses to rFPV-VP2 in mouse serum

Group	Number	Day post inocluation				
		0	14	21	35	49
NC	1#	0.105	0.138	0.145	0.184	0.134
		0.198	0.198	0.198	0.176	0.156
		0.188	0.176	0.251	0.187	0.146
	2#	0.236	0.187	0.135	0.114	0.211
		0.145	0.11	0.156	0.135	0.145
		0.136	0.167	0.194	0.145	0.165
	3#	0.126	0.149	0.141	0.131	0.115
		0.138	0.129	0.162	0.134	0.167
		0.137	0.229	0.145	0.159	0.145
	4#	0.215	0.139	0.167	0.139	0.112
		0.149	0.187	0.182	0.199	0.163
		0.186	0.186	0.193	0.131	0.191
	5#	0.289	0.175	0.134	0.125	0.21
		0.228	0.158	0.117	0.111	0.145
		0.138	0.188	0.245	0.14	0.114
S-FPV-017	6#	0.144	0.176	0.156	0.231	0.211
		0.111	0.188	0.167	0.131	0.113
		0.182	0.165	0.178	0.148	0.135
	7#	0.191	0.187	0.183	0.176	0.156
		0.145	0.189	0.185	0.199	0.206
		0.177	0.176	0.056	0.124	0.187
	8#	0.199	0.175	0.166	0.156	0.189
		0.213	0.199	0.159	0.149	0.166
		0.111	0.167	0.111	0.125	0.177
	9#	0.139	0.21	0.145	0.156	0.187
		0.137	0.159	0.123	0.146	0.198
		0.119	0.149	0.156	0.113	0.213
	10#	0.287	0.124	0.156	0.159	0.137
		0.119	0.135	0.192	0.137	0.145
		0.13	0.135	0.114	0.148	0.166
rFPV-VP2	11#	0.134	0.326	0.982	1.532	1.956
		0.147	0.39	1.012	1.589	1.876
		0.188	0.376	1.135	1.597	1.932
	12#	0.198	0.392	1.151	1.687	1.888
		0.154	0.419	0.934	1.788	1.83
		0.197	0.424	0.965	1.989	1.865
	13#	0.188	0.477	0.954	1.666	1.943
		0.109	0.389	1.141	1.876	1.892
		0.287	0.366	1.114	1.654	1.843
	14#	0.281	0.486	0.929	1.765	1.953
		0.099	0.423	0.845	1.686	1.975
		0.197	0.345	0.978	1.654	1.908
	15#	0.125	0.389	1.175	1.778	2.108
		0.14	0.355	0.914	1.764	1.897
		0.188	0.375	0.899	1.876	1.765

Note: Three replicates were performed for each sample.
