# Supplementary material for: Construction and Immunogenicity Evaluation of a Recombinant Fowlpox Virus Expressing VP2 Gene of African Horse Sickness Virus Serotype 1
Source: Microorganisms. 2025 Dec 9;13(12):2807. doi: 10.3390/microorganisms13122807 (PMC12735409; doi:10.3390/microorganisms13122807)
Supplement: Supplementary file 1 [file microorganisms-13-02807-s001.zip › Supplementary Table S3.pdf]

Supplementary Table S3. Indirect ELISA detection of antibody responses to rFPV-VP2 in horse serum.

	Day post inocluation						
Number	0	20	50	64	78	108	119
	0.102	0.356	0.522	0.8	1.246	1.132	1.478
20#	0.133	0.331	0.568	0.867	1.339	1.567	1.267
	0.146	0.344	0.514	0.913	1.157	1.446	1.478
	0.133	0.415	0.588	0.998	1.335	1.456	1.367
21#	0.129	0.371	0.571	0.976	1.346	1.475	1.588
	0.196	0.424	0.589	0.972	1.357	1.456	1.424
	0.106	0.336	0.612	0.996	1.425	1.445	1.578
31#	0.121	0.346	0.611	0.976	1.356	1.454	1.867
	0.151	0.325	0.633	0.999	1.4	1.458	1.543

Note: Three replicates were performed for each sample.
