# Supplementary figures and images for: Construction and Immunogenicity Evaluation of a Recombinant Fowlpox Virus Expressing VP2 Gene of African Horse Sickness Virus Serotype 1
Source: Microorganisms. 2025 Dec 9;13(12):2807. doi: 10.3390/microorganisms13122807 (PMC12735409; doi:10.3390/microorganisms13122807)

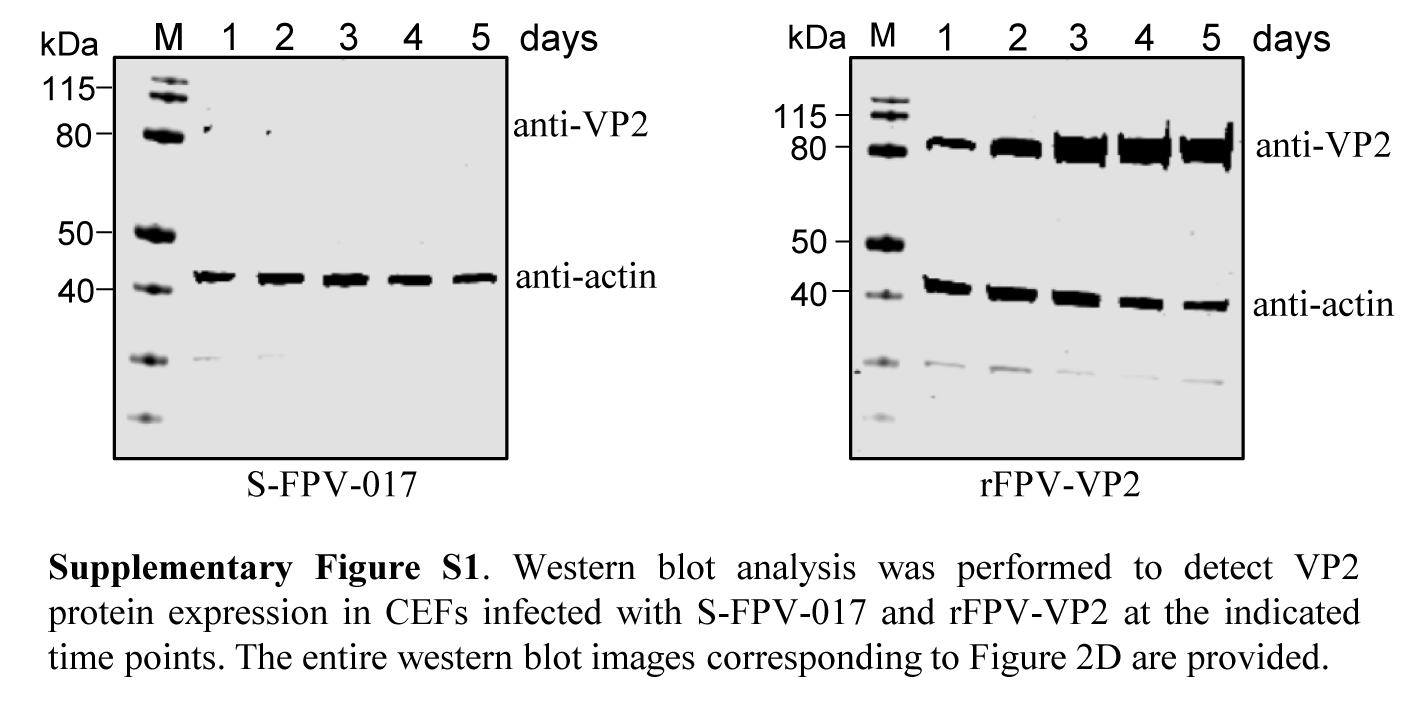

Supplement: Supplementary file 1 [file microorganisms-13-02807-s001.zip › Supplementary Figure S1.tif]
